# A novel *ex vivo* high-throughput assay reveals antiproliferative effects of idelalisib and ibrutinib in chronic lymphocytic leukemia

**DOI:** 10.18632/oncotarget.25419

**Published:** 2018-05-25

**Authors:** Daniel Primo, Lydia Scarfò, Aliki Xochelli, Mattias Mattsson, Pamela Ranghetti, Ana Belén Espinosa, Alicia Robles, Julian Gorrochategui, Joaquín Martínez-López, Javier de la Serna, Marcos González, Alberto Chaparro Gil, Eduardo Anguita, Sandra Iraheta, Veerendra Munugalavadla, Christophe Quéva, Stacey Tannheimer, Richard Rosenquist, Kostas Stamatopoulos, Joan Ballesteros, Paolo Ghia

**Affiliations:** ^1^ Vivia Biotech, Tres Cantos, Madrid, Spain; ^2^ Strategic Research Program on CLL and B Cell Neoplasia Unit, Università Vita-Salute San Raffaele and IRCCS Istituto Scientifico San Raffaele, Milan, Italy; ^3^ Institute of Applied Biosciences, Center for Research and Technology Hellas, Thessaloniki, Greece; ^4^ Department of Immunology, Genetics and Pathology, Science for Life Laboratory, Uppsala University, Uppsala, Sweden; ^5^ Department of Hematology, Hospital Universitario 12 de Octubre, Madrid, Spain; ^6^ Hematology Service, IBSAL-Hospital Universitario, Centro de Investigación del Cáncer (CIC)- IBMCC, Centro de Investigación Biomédica en Red de Cáncer (CIBERONC), Universidad de Salamanca, Salamanca, Spain; ^7^ Department of Hematology, Hospital Clínico San Carlos, Instituto de Investigación Sanitaria San Carlos (IdISSC), Madrid, Spain; ^8^ Department of Hematology and Hemotherapy, Hospital Universitario de Canarias, La Laguna, Spain; ^9^ Gilead Sciences, Foster City, CA, USA; ^10^ Department of Molecular Medicine and Surgery, Karolinska Institutet, Stockholm, Sweden; ^11^ Department of Medicine, Universidad Complutense de Madrid (UCM), Madrid, Spain

**Keywords:** chronic lymphocytic leukemia, Ibrutinib, idelalisib, antiproliferative, ex vivo

## Abstract

PI3Kδ (idelalisib) and BTK (ibrutinib) inhibitors have demonstrated significant clinical activity in chronic lymphocytic leukemia (CLL) interfering with the cross-talk between CLL cells and the lymph node microenviroment, yet their mechanism of action remains to be fully elucidated. Here, we developed an *ex vivo* model with the aim of reproducing the effects of the microenvironment that would help shed light on the *in vivo* mechanism of action of idelalisib and ibrutinib and predict their clinical efficacy in individual patients. First we explored the effects of various cell-extrinsic elements on CLL apoptosis and proliferation and found that the combination of CpG+IL2+HS5 stromal cell line + human serum +CLL plasma and erythrocyte fractions represented the best co-culture conditions to test the effects of the novel inhibitors. Then, using this assay, we investigated the impact of idelalisib and ibrutinib on both survival and proliferation in 30 CLL patients. While both drugs had a limited direct pro-apoptotic activity, a potent inhibition of proliferation was achieved at clinically achievable concentrations. Notably, up to 10% of CLL cells still proliferated even at the highest concentrations, likely mirroring the known difficulty to achieve complete responses *in vivo*. Altogether, this novel assay represents an appropriate *ex vivo* drug testing system to potentially predict the clinical response to novel inhibitors in particular by quantifying the antiproliferative effect.

## INTRODUCTION

The microenvironment critically promotes the development and progression of CLL, favoring leukemic cell survival and proliferation as well as inducing drug resistance [1–3]. Therefore, in order to predict more reliably the actual clinical (i.e. *in vivo*) response to drugs by analyzing primary CLL cells *ex vivo,* it is essential to develop assays that would take into account the key role of the microenvironment as well as the pharmacology of the drugs.

Several co-culture systems have been used in the past to simulate the *in vivo* microenvironment aiming to reproduce the proliferative signals of the so-called “proliferation centers” [4–10]. Combinations of cytokines and soluble molecules, such as CD40L+CpG [11], CD40L+IL21 [12], CpG+IL2 [13, 14], alone or in combination with stromal cell lines [11, 15], have been used to reproduce the stimuli provided by the microenvironment, however, definitive conclusions have not been reached. The need for more appropriate *ex vivo* assays is particularly relevant nowadays in order to assess the actual mechanism of action of novel targeted drugs such as the B cell signaling inhibitors idelalisib (a PI3K-δ inhibitor) [16, 17] and ibrutinib (a BTK inhibitor) [18–22] and predict *ex vivo* the anti-leukemic effect that may be achieved *in vivo*. These drugs act through distinct mechanisms compared to standard chemotherapy and monoclonal antibodies that are not yet fully understood but appear to be critically dependent on the surrounding microenvironment [16, 23, 24]. These inhibitors have already shown effects both *in vitro* and *in vivo* on cell survival, migration and proliferation [17–19, 25]. When administered to patients, both drugs appear to have a limited capacity to elicit cell apoptosis; indeed, using the current *ex vivo* assays, a proapoptotic effect on CLL cells is seen only at high micromolar concentrations difficult to attain *in vivo* [16, 26]. On the contrary, apoptosis may be indirectly induced through the typical mobilization effect of the leukemic cells from the tissues to the blood, depriving the cells of the supportive action of the tissue microenvironment [20, 27, 28]. That notwithstanding, this cannot fully explain the sustained responses achieved with both drugs. Hence, it remains to be explained how and why these drugs are so beneficial when administered to patients.

In this study, we aimed at designing and validating an *ex vivo* co-culture system that simulates more closely the conditions present in the leukemic tissue microenvironment and enable not only CLL cell survival but also proliferation *ex vivo*. Such system would reproduce more accurately what is actually happening *in vivo* within the lymph nodes and, in particular, the events within the proliferation centers that are considered the reservoir of the disease. The herein described *ex vivo* assay incorporating microenvironmental stimuli enabled us to identify an anti-proliferative activity for idelalisib and ibrutinib that may underlie, at least in part, the effects of both drugs *in vivo*.

## RESULTS

### Assessing *ex vivo* the impact of different combinations of stimuli on CLL apoptosis and proliferation

In order to reproduce more closely the complexity of the *in vivo* microenvironment in CLL, we explored the effects of various microenvironmental mimicking elements on CLL cell survival and proliferation.

We previously reported that the native environment (NE), defined as the plasma and erythrocyte/granulocyte fraction of a Ficoll gradient is critical to prevent artifacts in *ex vivo* drug testing [29] offering higher predictive accuracy regarding clinical responses against acute myeloid leukemia [Montesinos *et al*, manuscript in preparation]. On these grounds, we explored the effect of the NE on CLL samples by testing pooled NE from 5 different healthy donors (HD) or 5 CLL patients on 11 cryopreserved CLL samples from 5 IGHV-unmutated (U-CLL), 5 IGHV-mutated (M-CLL) and 1unknown. The decision to pool NE from different individuals was taken in order to minimize interpatient or interdonor variability. The median % of non-apoptotic cells after thawing was 62% (ranging 16–92).

**Figure 1:**
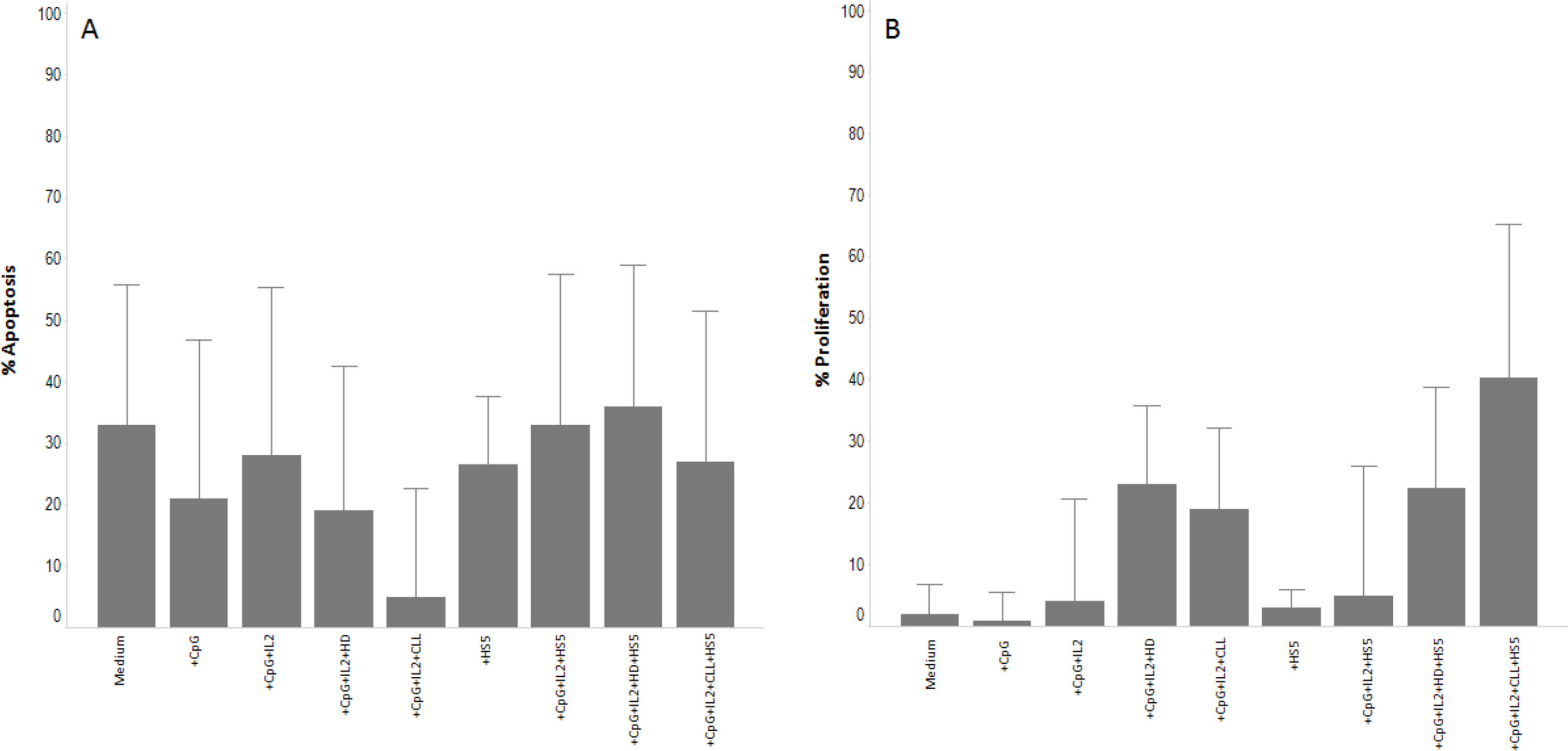
Effect of the NE on CLL apoptosis and proliferation Histograms showing the effect on apoptosis and proliferation of the NE from 5 CLL or 5 HD in 11 cryopreserved CLL samples. (Panel **A**) show the apoptotic effect of the CpG+IL2 cocktail in presence of the different NE or HS5 cell line. (Panel **B**) show the proliferation effect of the CpG+IL2 cocktail in presence of the different NE or HS5 cell line. Data from apoptosis are subtracted from the value of apoptosis after thaw. Each condition was acquired in triplicate and data represent the median ± SD.

With CpG and IL2 stimulation as backbone, given the well known proliferative effect on CLL leukemic cells [14], we found that the addition of NE, from HD or CLL patients decreased apoptosis (median % of apoptotic cells without NE vs NE from HD vs NE from CLL: 28 ± 27 vs 19 ± 23 vs 5 ± 17; *p* < 0.001), while the addition of the stroma cell line HS5 abrogated the positive effect of the NE resulting in similar protection from apoptosis in all 3 conditions (35%) (Figure 1A). A different trend was observed regarding proliferation: in the presence of HS5, CLL cells exposed to NE from CLL showed a marked increase of the proliferation rate (median % of proliferating cells without NE vs NE from HD vs NE from CLL: 5 ± 21 vs 22 ± 21 vs 40 ± 25) (Figure 1B). Therefore, the best combination of microenvironmental mimicking factors was achieved when including the NE from CLL patients. We also compared Human Serum (HS) 10% vs FBS 10% in 10 CLL samples in the presence of the NE from CLL patients and HS5, and observed that HS exerted a superior protective effect against apoptosis (median % of apoptotic cells with HS 10% vs FBS 10%: 48 ± 28 vs 65 ± 19, *p* = 0.004). No significant differences were observed between U-CLL and M-CLL in this set of experiments. Based on these results, we decided to include NE from CLL and HS in all future assessments.

Capitalizing on the capacity of the PharmaFlow platform (formerly called ExviTech) to screen multiple conditions simultaneously [29], we next examined different combined microenvironmental mimicking triggers for their effects on CLL cells. To this purpose, cryopreserved PB samples from 7 U-CLL and 5 M-CLL CLL cases were tested for multiple conditions using 2 additional backbone stimulations previously reported as effective in improving CLL proliferation and survival, besides CpG+IL2 [13, 14], namely sCD40L+CpG [11] and sCD40L+IL21 [12], along with the NE pooled from PB of CLL samples and HS (Table 1). The median % of non-apoptotic cells after thawing was 88% (ranging 73–97). Then, in a stepwise fashion, we added to each of these combinations one or more of the following elements: BAFF, sCD40L, BcR stimulation (anti-IgM) and the HS5 stromal cell line.

**Table 1:** List of cytokine combinations tested to compare viability and proliferation

No signal	CD40L+CpG Signalling	CD40L+IL21 Signalling	CpG+IL2 Signalling
Medium	CD40L+CpG	CD40L+IL21	CpG+IL2
Medium+CLL NE	CD40L+CpG+Ig	CD40L+IL21+Ig	CpG+IL2+CD40L
Medium+HS5	CD40L+CpG+BAFF	CD40L+IL21+BAFF	CpG+IL2+Ig
Medium+CLL NE+HS5	CD40L+CpG+IL21	CD40L+IL21+HS5	CpG+IL2+HS5
	CD40L+CpG+HS5	CD40L+IL21+Ig+HS5	CpG+IL2+BAFF
	CD40L+CpG+Ig+HS5	CD40L+IL21+BAFF+HS5	CpG+IL2+CD40L+HS5
	CD40L+CpG+BAFF+HS5		CpG+IL2+Ig+HS5
	CD40L+CpG+IL21+HS5		CpG+IL2+BAFF+HS5

CLL: Chronic Lymphocytic Leukemia; NE: Native Environment.

**Figure 2:**
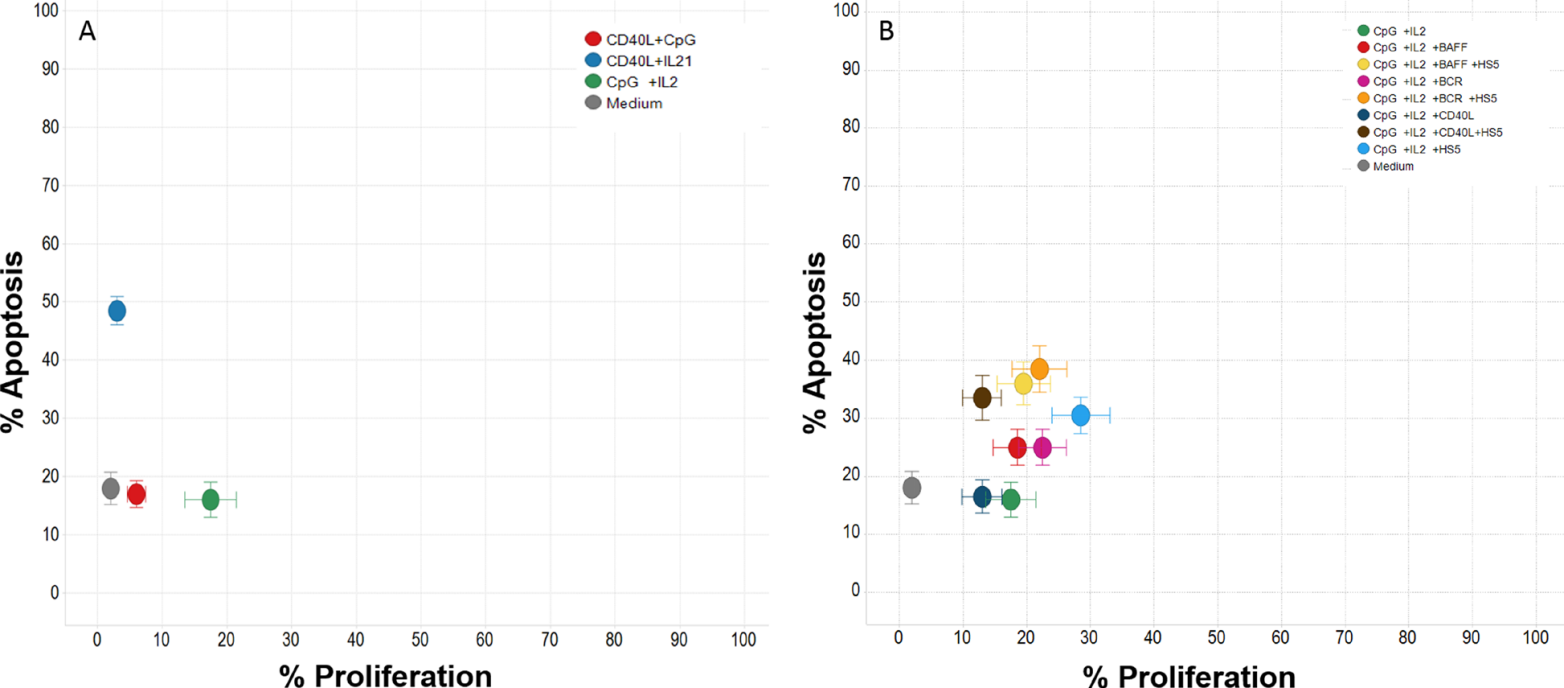
Effect of different cytokines on CLL apoptosis and proliferation Twelve progressive CLL frozen samples (7 IGHV mutated and 5 unmutated) were used to test the effect of 26 different cytokine conditions with media containing a pool of 3 progressive CLL NE and human serum. (Panel **A**) shows the results of the 3 backbone stimulation (sCD40L+CpG, sCD40L+IL-21 and CpG+IL-2) in terms of apoptosis and proliferation reflecting that CpG+IL2 provided better results. (Panel **B**) shows the incorporation of additional stimuli (BAFF, anti-BCR, sCD40L, HS5) to the CpG+IL2 backbone stimulation reflecting that the use of CpG+IL2 and HS5 (at 1:100 ratio) showed the best effects with a median of 30 ± 19% of apoptosis and 30 ± 27% of proliferation. Data from apoptosis are subtracted from the value of apoptosis after thaw.

Of all specific backbone stimulation tested, CpG+IL2 provided better results in terms of protection from apoptosis and proliferation vs sCD40L+CpG or sCD40L+IL21 (Figure 2A). Incorporation of additional stimuli (BAFF, anti-BCR, sCD40L, HS5) to the CpG+IL2 combination consistently improved proliferation, however at a cost of increased apoptosis (Figure 2B), with the notable exception of the concomitant use of CpG+IL2 and HS5 (at 1:100 ratio) that showed the best effects with a median of 30 ± 19% of apoptosis and 30 ± 27% of proliferation in the 12 cryopreserved progressive CLL samples analyzed (Table 2 and Supplementary Figure 1). The lack of improvement in terms of proliferation when cells were stimulated through CD40 was confirmed in additional experiments using other soluble or multimeric sCD40L in the cocktail. Interstingly, in this co-culture model, HS5 cell line already express CD40L (Supplementary Figure 2) that could overcome the effect by exogenous addition of the soluble forms.

**Table 2:** Percentage of apoptosis and proliferation of the cytokine combinations tested in 12 CLL

Cytokine	% Apoptosis	% Proliferation
Medium	18 ± 17	2 ± 1
Medium+CLL NE	17 ± 14	2 ± 1
Medium+HS5	27 ± 17	2 ± 2
Medium+CLL NE+HS5	21 ± 15	2 ± 3
CD40L+CpG	17 ± 14	6 ± 8
CD40L+CpG+Ig	23 ± 14	5 ± 7
CD40L+CpG+BAFF	19 ± 13	7 ± 8
CD40L+CpG+IL21	49 ± 19	6 ± 11
CD40L+CpG+HS5	30 ± 19	5 ± 13
CD40L+CpG+Ig+HS5	36 ± 20	4 ± 12
CD40L+CpG+BAFF+HS5	39 ± 22	6 ± 12
CD40L+CpG+IL21+HS5	62 ± 20	10 ± 18
CD40L+IL21	48 ± 14	3 ± 3
CD40L+IL21+Ig	51 ± 15	2 ± 1
CD40L+IL21+BAFF	52 ± 19	2 ± 4
CD40L+IL21+HS5	61 ± 16	3 ± 3
CD40L+IL21+Ig+HS5	62 ± 15	4 ± 4
CD40L+IL21+BAFF+HS5	64 ± 19	3 ± 3
CpG+IL2	16 ± 18	18 ± 24
CpG+IL2+CD40L	16 ± 17	13 ± 19
CpG+IL2+Ig	25 ± 19	19 ± 22
CpG+IL2+BAFF	25 ± 18	19 ± 22
CpG+IL2+HS5	30 ± 19	30 ± 27
CpG+IL2+CD40L+HS5	33 ± 23	14 ± 17
CpG+IL2+Ig+HS5	38 ± 24	23 ± 25
CpG+IL2+BAFF+HS5	36 ± 22	20 ± 25

Data from apoptosis are subtracted from the value of apoptosis after thaw. Results are represented as median ± SD (*N* = 12).

Although, overall, CpG+IL2+HS5 proved to be the best combination, still it showed significant heterogeneity between samples regarding the protection from apoptosis (range, 7–70%) and induction of proliferation (range, 1–91%). We investigated if this could be associated with particular, prognostically relevant, biological features of each CLL sample such as IGHV gene somatic hypermutation status, CD38 expression, and cytogenetic abnormalities detected by FISH. We found that U-CLL cases showed a significantly more prominent proliferative response (48 ± 27% vs 17 ± 14% for M-CLL; *p* < 0.001); however, no significant differences were evident in terms of apoptosis, although U-CLL cases showed a trend for more homogeneous protection (25 ± 19 vs 38 ± 19 in U-CLL vs M-CLL cases; *p* = 0.5).

### Idelalisib and ibrutinib induce potent inhibition of B cell proliferation

Based on the previous results, the final combination, i.e. CpG+IL2+HS5 (1:100) + HS 10% + pooled CLL NE, was used as an *ex vivo* assay in order to test the effect of idelalisib and ibrutinib on 30 cryopreserved samples from CLL patients in need of treatment (Table 3). We analyzed responses to both drugs after 96 h; dose concentrations ranged from 150 μM to 0.54 nM to cover a wide window of concentrations.

**Table 3:** Clinical features of the CLL patients included to test idelalisib and ibrutinib in the study

Patient Code	Gender	Age	Stage (Binet)	IGHV SHM status	FISH	CD38	Subsequent need of therapy	Previous Treatment
1	Male	51	B	Unmutated	Normal	Negative	Yes	No
2	Male	54	A	Unmutated	del(11q)	Positive	Yes	Yes
3	Male	81	C	Unmutated	del(13q), del(17p)	Positive	Yes	No
4	Male	53	C	Mutated	del(13q)	Positive	Yes	Yes
5	Male	64	B	Mutated	del(17p)	Negative	Yes	No
6	Female	68	A	Mutated	Normal	Negative	Yes	No
7	Female	75	A	Unmutated	N/A	Negative	Yes	No
8	Male	69	C	Unmutated	del(17p)	Negative	Yes	Yes
9	Female	72	B	Unmutated	Trisomy12	Negative	Yes	No
10	Male	59	C	Mutated	Trisomy12	N/A	Yes	Yes
11	Male	61	A	Mutated	del(11q)	Negative	Yes	Yes
12	Male	44	A	Mutated	del(13q)	Negative	Yes	Yes
13	Male	69	A	Unmutated	N/A	Positive	Yes	Yes
14	Male	76	A	Unmutated	del(11q)	Positive	Yes	Yes
15	Male	67	A	Mutated	Normal	Negative	Yes	Yes
16	Male	80	A	Unmutated	Trisomy12	Positive	No	No
17	Female	87	A	Mutated	N/A	Negative	Yes	Yes
18	Female	74	A	Unmutated	del(11q)	Negative	Yes	Yes
19	Male	66	A	Unmutated	del(17p)	Negative	Yes	Yes
20	Female	80	B	N/A	Trisomy12	Positive	Yes	Yes
21	Male	84	C	Mutated	del(13q)	Negative	Yes	No
22	Female	32	B	Unmutated	Normal	Negative	Yes	Yes
23	Female	73	A	N/A	del(13q)	Negative	Yes	Yes
24	Female	61	A	Unmutated	del(17p)	Positive	Yes	Yes
25	Male	42	A	Unmutated	del(13q)	N/A	Yes	N/A
26	Female	75	A	Unmutated	del(11q)	Positive	Yes	N/A
27	Male	47	A	Unmutated	N/A	N/A	Yes	N/A
28	Male	57	A	Mutated	Normal	Positive	No	No
29	Male	66	C	Mutated	del(13q)	Negative	Yes	No
30	Male	63	B	Unmutated	del(17p)	Negative	Yes	No

N/A: Not analyzed.

**Figure 3:**
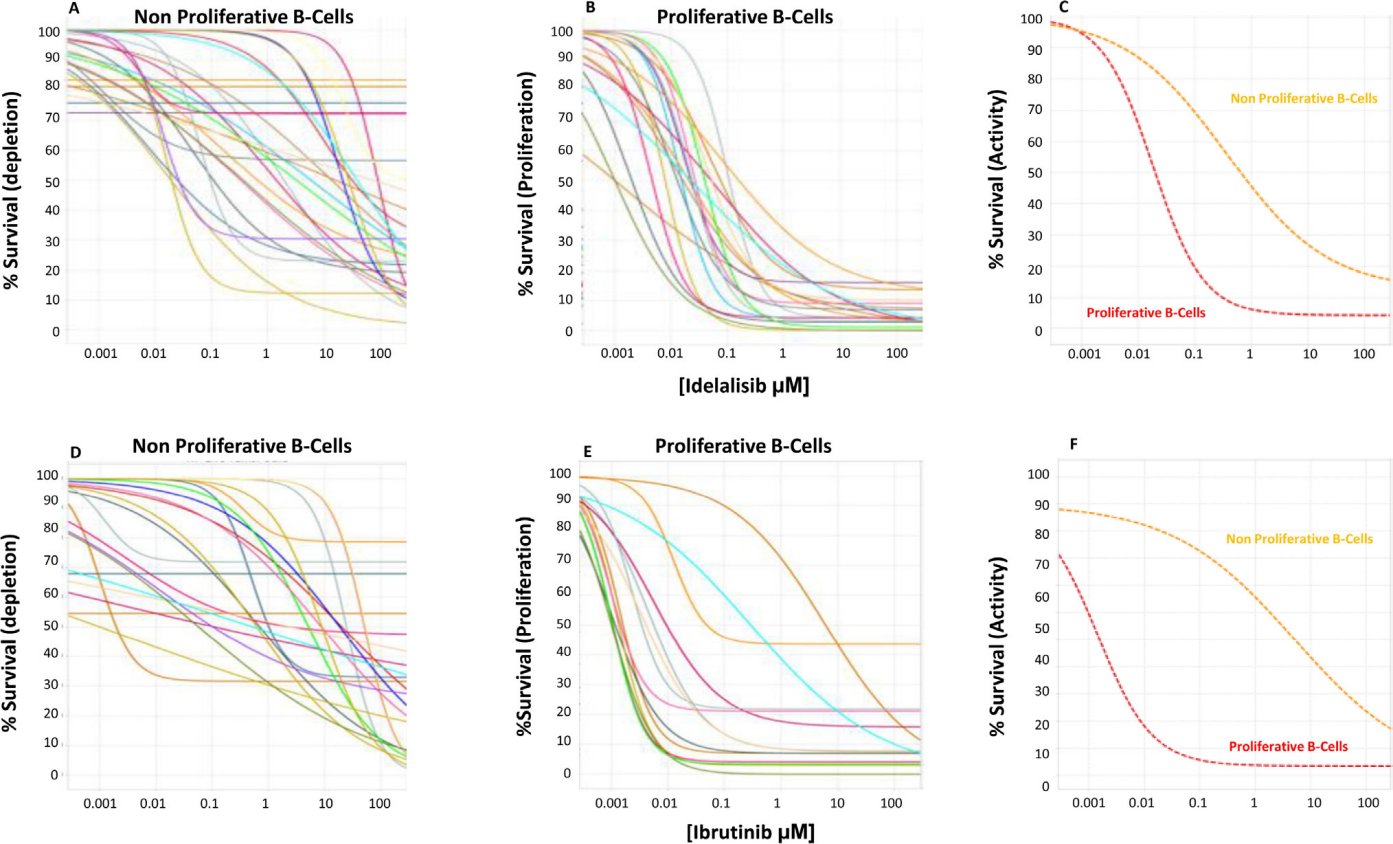
Idelalisib dose exposure evaluation Twenty-nine progressive CLL frozen samples (represented with differently colored lines) were tested at 96h with the CpG+IL2+HS+CLL NE media for dose response of idelalisib (Panels **A**–**C**) and ibrutinib (Panels **D**–**F**) in both the non-proliferative (panel A, D) and proliferative (panel B, E) fractions, measuring the % of live leukemic cells at each concentration shown as % Survival. We found little effect on the non-proliferative CLL fraction, suggesting a limited pro-apoptotic depletion activity of the drugs. In contrast, potent inhibition of proliferation with median potency (EC_50_) of 28 nM for idelalilisib and 550 nM for ibrutinib was observed. The efficacy was nearly complete leaving a median of 5% and 8% resistant CLL cells that proliferated at the highest doses of idelalisib or ibrutinib. (Panel C) (idelalisib) and (Panel F) (ibrutinib) represents the media of the effect in the non-proliferative CLL cells (orange line) and the proliferative B-fraction (red line) showing a predominant antiproliferative activity of both drugs.

**Figure 4:**
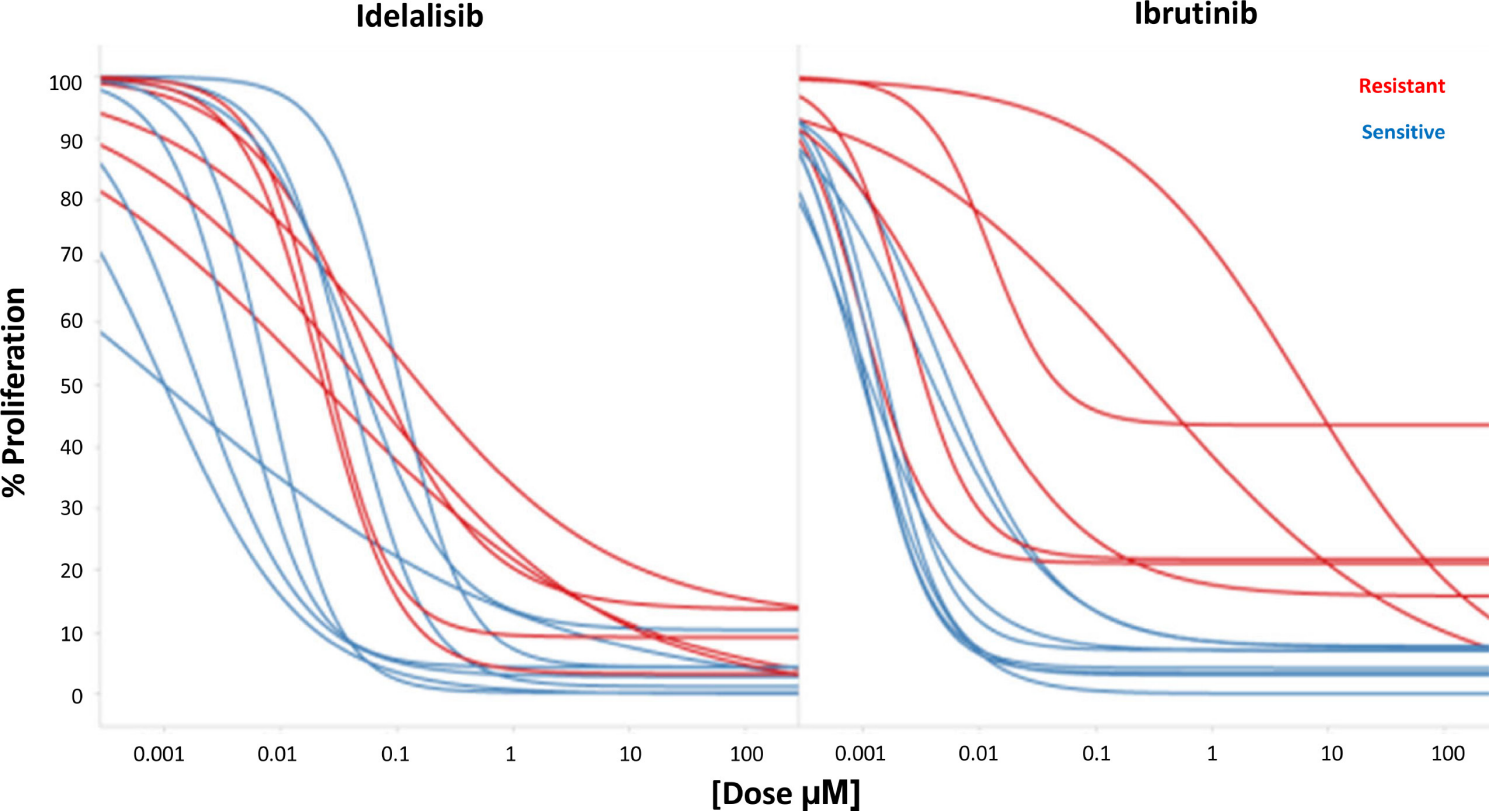
Simultaneous evaluation of Idelalisib and Ibrutinib Cross reactivity of ibrutinib and idelalisib according to their *ex vivo* pharmacological profile. Red samples represent the ibrutinib resistant samples.

In comparison to the co-culture condition where the number of cells increased due to augmented proliferation and decreased apoptosis, when cells where incubated in the presence of both idelalisib (*n* = 29) and ibrutinib (*n* = 25), the number of CLL cells was significantly affected in a dose-dependent manner. In order to better understand the basis for these changes we analyzed proliferating vs non-proliferating fractions of CLL cells separately (Figure 3), and showed that activity for idelalisib and ibrutinib (EC_50_ of 13.7 ± 30.3 μM and 13 ± 21.2 μM) was overall evident on both fractions. A more detailed analysis showed that both idelalisib and ibrutinib led to a limited induction of apoptosis that was restricted to the non-proliferating fraction (EC_50_ of 12.5 ± 22.5 μM and 28.3 ± 99 μM, respectively). In contrast, a potent and selective inhibition of proliferation was evident (average EC_50_ of 0.03 ± 0.03 μM for idelalisib and 0.55 ± 1.6 μM for ibrutinib), though the anti-proliferative effect of either drug was never complete, with an average of 5.2 ± 5% and 8.8 ± 11.7 CLL cells, respectively, remaining in active proliferation even at the highest doses of either drug. High interpatient variability was apparent in the effect on proliferating cells, with distinct patterns for each drug. Ibrutinib showed 6/25 outlier samples displaying a lower *ex vivo* sensitivity relative to most of the other samples; 3 samples showed a substantially higher potency (EC_50_) thus contrasting the other 3 samples where a substantially higher efficacy was noted (E_max_) (red-marked in Figure 4). Idelalisib showed a more homogenous pattern with few outliers. As a result, there was a higher variability in the standard deviation of ibrutinib vs idelalisib in the average values for potency (1.6 vs 0.03 μM) and efficacy (11.7 vs 5.2%).

To further study these different profiles, we compared effects in 14 samples where both drugs could be tested simultaneously *ex vivo* (Figure 4), including the 6 previously mentioned ‘ibrutinib outliers’ plus 8 additional cases. As expected, the 6 ‘ibrutinib outliers’ cases showed a significant higher Area Under the Curve (AUC) for ibrutinib compared to the remaining 8 cases (3503 ± 1616 vs 747 ± 418, *p* < 0.001), suggesting lower *ex vivo* sensitivity to the drug. Interestingly, the latter 8 samples did not show any outlier with low sensitivity to idelalisib, although higher AUCs were also observed (1423 ± 767 vs 535 ± 513, *p* = 0.02; Figure 4).

Next, we analyzed if the *ex vivo* sensitivity to either idelalisib or ibrutinib could be associated to the biological profile of the studied cases (Table 4). No significant association was identified with any evaluated parameters [clinical stage, IGHV gene somatic hypermutation status, CD38 expression, trisomy 12, del(13q), del(11q), del(17p)]. Surprisingly, isolated del(13q), traditionally considered as prognostically favorable, was associated with a worse *ex vivo* response as evidenced by a significant higher AUC for both idelalisib (1670 vs 577, *p* = 0.01) and ibrutinib (3507 vs 1174, *p* = 0.02). These results indicate that both idelalisib and ibrutinib are effective in inhibiting CLL cell proliferation *ex vivo* even in cases with adverse biological background and, moreover, that this assay may help identifying the drug to which each case may best respond.

**Table 4:** Ibrutinib and idelalisib pharmacological profile in the proliferative fraction according to their clinical and biological parameters

		Idelalisib			Ibrutinib		
		AUC	*N*	*P*-value	AUC	*N*	*P*-value
Stage	A	1002	8	0.71	2257	8	0.50
	B+C	830	5		1664	5	
IGHV status	Mutated	1049	5	0.74	2519	5	0.40
	Unmutated	894	8		1669	8	
CD38 expression	≥20%	504	5	0.12	1179	5	0.18
	≤20%	1206	8		2560	8	
Trisomy 12	Positive	213	2	0.18	522	2	0.26
	Negative	1040	11		2141	11	
del(13q)	Positive	1670	4	0.01	3507	4	0.02
	Negative	577	9		1174	9	
del(11q)	Positive	596	3	0.45	696	3	0.20
	Negative	1008	10		2251	10	
del(17p)	Positive	850	2	0.90	1727	2	0.86
	Negative	926	12		1962	12	

## DISCUSSION

Novel drugs, such as, ibrutinib [30–32], idelalisib [33–35] and venetoclax [36], have been recently approved for clinical use in CLL, while many other drug candidates are currently in late-stage clinical development [37–39]. In most instances, these novel compounds act through mechanisms different from classic chemo-immunotherapy, targeting fundamental pathophysiologic mechanisms and processes, e.g. signaling pathways which mediate microenvironmental interactions critical for CLL cell survival and proliferation. Despite the remarkable efficacy of the signaling inhibitors approved for clinical use, i.e. the PI3Kδ inhibitor idelalisib and the BTK inhibitor ibritinib, several issues remain unresolved, especially regarding their precise mode of interaction with the microenvironment. This may be relevant for understanding why they are generally unable to lead to complete responses (CR) and, even if CR is achieved, to eradicate minimal residual disease. Such comprehension has been hampered by the lack of suitable *ex vivo* pharmacological assays that may allow to recapitulate *ex vivo* the *in vivo* action of these new agents. Such assays may also be useful to predict *in vivo* efficacy, personalize treatment and test potential combinations for overcoming resistance.

Our study revealed that factors within the NE, i.e. Ficoll fractions of plasma and red blood cells (RBC)/granulocytes, from the peripheral blood of patient samples, greatly protect CLL cells from apoptosis *ex vivo*. The latter could be further improved by the addition of the stroma cell line H5S and HS. Pooled CLL plasma were used to avoid interpatient variability, however future experiments should be performed to understand in detail the contributing factors affecting apoptosis induction and/or proliferative capacity. In order to better mimick the *in vivo* microenvironment, and in particular to elicit cell proliferation as it occurs *in vivo* in the tissue proliferation centers, we also added cytokine combinations previously shown to be effective in increasing the fraction of leukemic cells in cycle. CpG and IL2 showed the best proliferative effect in the presence of the NE, stroma and human serum with a limited impact on cell survival, thus, collectively representing the best ‘cocktail’ for concomitantly studying proliferation and survival of CLL cells *ex vivo*. Contrary to previous reports [40–42], adding additional stimulatory B cell factors individually did not improve the overall assay conditions, suggesting that these factors may already be present and thus replaced in the CLL NE.

Altogether, our approach enabled developing an engineered microenvironment more realistically simulating the *in vivo* setting. Hence, the assay described here seems more appropriate for both *ex vivo* drug testing and *ex vivo* predicting the responses potentially achievable when administering the drug *in vivo*. Thanks to this assay, we were able to show that idelalisib has an anti-proliferative effect on CLL cells, as recently suggested [43]. Similarly, we were also able to confirm the previous observation that ibrutinib targets CLL cell proliferation with very limited if any pro-apoptotic effect [19, 44]; this supports that the results obtained in our novel assay for *ex vivo* drug testing are reliable. To further underscore the robustness of the assay, it should be noted that apoptosis was initially described in the case of ibrutinib or other signaling inhibitors (e.g. SYK inhibitors) only when these drugs were tested *ex vivo* on CLL cells in the absence of stromal support and/or at much higher concentration not attainable *in vivo* [45–49]. This does not contradict the fact that, *in vivo*, the typical mobilization effect of this novel class of drugs may indirectly favor apoptotic death due to the deprivation of prosurvival stimulation from the tissue microenvironment, including the stroma. More generally, the limited *ex vivo* propapoptotic effect may be reflected in the persistent lymphocytosis that may last for over a year [50–52].

Noticeably, the herein reported assay allows to identify patients where either drug may achieve less antiproliferative activity. In the case of ibrutinib, a few outlier samples were identified that required much higher doses to achieve an antiproliferative effect (EC_50_ or potency); in the case of idelalisib, a higher percentage of proliferating cells persisted at very high doses compared to ibrutinib. If these differences are found to translate into differential clinical activity validated in a larger patient cohort, they may provide a future basis for selection between these drugs by simply screening peripheral blood samples prior to treatment initiation.

More importantly, neither drug was found to completely eliminate all CSFE^low^ proliferating cells in the tested cases. Indeed, a variable number of proliferating leukemic cells remained following either ibrutinib or idelalisib treatment, likely explaining the limited capacity of either drug to induce complete and profound responses [32, 34]. In addition, this might indicate the existence of a fraction of CLL cells which are inherently resistant to these drugs. Whether such cells may be specifically and effectively targeted by drug combinations needs to be experimentally determined, potentially by capitalizing on the ability of our novel *in vitro* assay to pre-clinically test in a systematic fashion different plausible combinations. It will also be interesting to further exploit this assay to investigate if the CLL cases in which a larger fraction of cells appear to be non-responsive to the anti-proliferative effect of ibrutinib and idelalisib are actually those achieving less optimal clinical responses and if the depth of the response obtained *in vivo* may correlate to the percentage of cells responding *ex vivo*.

In conclusion, the herein described novel assay, enabling high-throughout exploration of combinations of drugs as well as real-time identification and separation of proliferating/non-proliferating cells, is expected to assist in predicting *ex vivo* the response to single or multiple drugs in each individual CLL patient requiring treatment. Although this needs to be tested prospectively, it opens up the potential of tailoring the drug or the dose to each individual patient.

## MATERIALS AND METHODS

### Patient samples and Ethics statement

Peripheral blood samples from 47 patients diagnosed with CLL according to the iwCLL guidelines [53] were obtained following informed consent in accordance with the Declaration of Helsinki. The study was approved by the local ethical committee of the participating centers. Table 1 shows the clinical parameters collected for each patient.

Peripheral blood mononuclear cells were separated by Ficoll Histopaque 1077 (Sigma-Aldrich, St. Louis, MO) density gradient and subsequently cryopreserved in fetal bovine serum (FBS; Thermo Fisher Scientific, Waltham, MA) plus 10% dimethylsufoxide in liquid nitrogen until further analysis. After separation by density gradient, the plasma and the RBC from the peripheral blood of HD and CLL samples were also recovered. The plasma fractions were stored at −80°C until use. RBCs were kept at 4°C for a maximum of 35 days with the addition of the anticoagulant citrate phosphate dextrose adenine solution (CPDA-1; Terumo Corporation, Tokyo, Japan) (150 μl CPDA/ml RBCs). These two fractions (plasma and RBCs) were added in combination to the cell culture media during the experimental procedures and referred to as NE.

### *In vitro* cell culture

Frozen CLL samples were thawed and cultured in 96-well plates at a concentration of 10 × 10^6^ cells/ml in AIM-V AlbuMAX culture media that better support B-cell survival (Invitrogen, Carlsbad, CA) [54]. In order to test the best culture conditions in terms of proliferation and survival of leukemic cells, the following factors (either alone or concomitantly) were added to the wells:

FBS or HS (Sigma-Aldrich), at 10% or 20% concentration, 2% HEPES, 1% Zell Shield antibiotic (Labclinics, Barcelona, Spain), and 1% L-glutamine 200 mM (Lonza, Hopkinton, MA).

Cytokines/soluble molecules, in double or triple combinations: IL2 (Peprotech, London, United Kingdon), at 50 ng/ml; IL21 (Peprotech, London, United Kingdon), at 25 ng/ml; CpG ODN (ODN2006; Miltenyi Biotech, Bergisch Gladbach, Germany), at 1 μg/ml; sCD40L (Peprotech, London, United Kingdon), at 1 μg/ml; sCD40L plus enhancer (Enzo Life Sciences, Farmingdale, NY, USA), at 1 μg/ml; BAFF (Peprotech, London, United Kingdon), 10 ng/ml; anti-IgM+anti-IgG antibodies (Vitro, Sevilla, Spain), at 10 μg/ml.

HS5 stromal cell line (ATCC^®^ CRL-11882™) was purchased from the American Type Culture Collection (ATCC; Rockville, MD). Cells were maintained at 37°C in 5% CO_2_ atmosphere in Dulbecco´s Modified Eagle´s Medium (DMEM; Sigma) supplemented with 2mM L-Glutamine, 1% penicillin/streptomycin (Sigma-Aldrich) and 10% heat-inactivated FBS. Early passages of the cell line were used for the experiments. Cells were plated in 96-well plates and incubated for 24 hours to allow cells to adhere. 1 × 10^4^ CLL cells were then cultured at a ratio of 100:1 (B-Cells:HS5) on confluent layers of the stroma.

NE; plasma and RBC of NE were also added in combination at a concentration of 1 μl/60 μl. 0,5 μl from a pooled RBC plus 0,5 μl from a pooled plasma were added in a final 60 μl volume.

Cultures were harvested after 96h for survival and proliferation analysis.

### Apoptosis and viability assays

To lyse RBCs, 180 mL of ammonium chloride lysis solution was added to each well (2 g KHCO_3_, 16.58 g NH_4_Cl, 0.074 g Na_2_-ethylenediaminetetraacetic acid [EDTA] 2H_2_O, H_2_O to 1 L). Following 10 minute incubation at 4°C, each plate was centrifuged for 5 minutes at 1200 rpm and the supernatant removed. The lysis step was performed twice. For staining, 20 μl of a combination of annexin-V CF Blue (Immunostep, Salamanca, Spain), CD19 PC7 (Clone J4.119, Beckman Coulter, Brea, CA) and CD45-APC (clon HI30, Immunostep) resupended in binding buffer (BB) (2.4 g HEPES, 8.19 g NaCl, 0.37 g Cl_2_Ca, H_2_O to 1 L) were added. After 15 minutes of incubation at room temperature in the dark, a wash step was performed using BB solution. The pellet was resuspended in 80 μL BB for cytofluorimetric analysis utilizing PharmaFlow platform, as previously reported [29]. Apoptotic cells was measured as the percentage of Annexin V positive cells subtracting the value of the apoptotic cells after thaw. In selected experiments, 7-amino-actinomycin (7-AAD) (BD Biosciences) was added to confirm the Annexin-V results (See Supplementary Figure 3).

### Proliferation assay

The Vybrant^®^ CFDA SE Cell Tracer Kit (Invitrogen) was used to measure cell proliferation. The CFDA SE (component A) was dissolved in dimethylsufoxide (component B) at a concentration of 5mM as stock solution and kept at −20°C until further use. CLL cells were adjusted at 10 × 10^6^ cells/ml in AIM-V AlbuMAX culture media without FBS. CFSE was added to a 1 ml cell suspension at a final concentration of 5 μM. After addition of CFSE, cells were vortexed and incubated at room temperature for 10 min with continuous shaking and light protected. At the end of the incubation period, the cells were resuspended in cold culture media with 10% FBS (complete culture media) and kept on ice for 5 min following two washes in cold complete culture media and maintained at 4°C until cytofluorimetric analysis in the PharmaFlow platform, as previously described [29]. Percentage of proliferation is defined as the percent of B-cells that have lost any level of CFDA labeling in the presence of exogenous stimuli.

### Drug assay activity testing

Cryopreserved CLL samples were diluted with AIM-V AlbuMAX (Invitrogen) supplemented with the NE, 10% of human serum (Sigma-Aldrich), 2% HEPES, 1% Zell Shield antibiotic (Labclinics), 1% L-glutamine 200 mM (Lonza), 1 μg/ml CpG ODN and 50 ng/ml IL2. This mixture was dispensed into 96-well plates containing the HS5 (100:1) cell line and transferred into a new 96-well plate containing idelalisib (Selleck Chemicals, Houston, TX) and ibrutinib (Selleck Chemicals). Drug plates were prepared using an Echo 550 Liquid Handler (LabCyte, Sunnyvale, CA). For each drug, 8 concentrations were used. The plates were incubated for 96 hours at 37°C in humidified air containing 5% CO_2_. Later, proliferation and viability were tested by flow cytometry in the PharmaFlow platform as indicated above.

Using the automated cell dispensing system Multidrop (Thermofisher), a precise constant number of leukemic cells is seeded in every well, all wells containing the same number of leukemic cells. This enables assessing compound activity by counting the absolute number of leukemic cells in each well by a proprietary automated flow cytometry platform described elsewhere [29], and comparing wells with a certain concentration of compound vs control wells without compound. Eight concentrations of each drug are used to calculate dose response curves to measure the compound activity. In order to identify live CLL cells, once the CLL cell subpopulation has been gated, Annexin V expression and FSC/SSC parameter is used to exclude apoptotic cells. Therefore, absolute counts of only non apoptotic leukemic cells is compared between different wells. This methodology is mechanistically unbiased measuring simultaneously in the same cell population both depletion and proliferaton of cells, as a decrease of non-proliferating cell counts (NP) or an increase in proliferating (PR) cell counts, respectively. Because we count only non apoptotic leukemic cells, we use the term Survival, normalized to the number of non apoptotic leukemic cells in the control cells as % Survival. This allows to plot in the same graph both proliferating and non-proliferating cells measured in the same well under the same conditions using the same cell counting method, as shown in Figure 3; it shows the difference in the dose response curves between NP vs PR cells using this same % Survival value in the Y axis.

Gate strategy and representative dot plots are provided (See Supplementary Figure 4) for the analysis of cell survival and proliferation.

### Statistical analysis

In order to determine the statistical significance of the differences observed between groups, the Mann-Whitney U and Wilcoxon tests were used. Drug potency and efficacy was estimated by a modelling approach to dose-response pharmacodynamics function. The model used (Equation 1) to fit the data was the most common single-site sigmoidal dose-response inhibitory model based on Hill equation, where the dependent variable (y) analyzed was the number of live CLL cells counted by flow-cytometry at every tested concentration of drug. Data points were fitted using the Levenburg Marquardt algorithm. Normalization of the results to calculate the survival index was performed referring each data point to the basal level parameter (E0). This approach allows the calculation of the normalized AUC value by integrating the curve function from the model between the lowest and highest concentrations.

$$y=A+\frac{B-A}{1+{\left(\frac{C}{x}\right)}^{D}}$$ (Equation 1)

A is the basal or E_0_, B is the E_max_, C is the EC_50_ and D the slope or Hill factor.

## SUPPLEMENTARY MATERIALS FIGURES



## Abbreviations

CLL: Chronic Lymphocytic Leukemia
NE: Native Environment
HD: Healthy Donors
HS: Human Serum
U-CLL: IGHV Unmutated CLL
M-CLL: IGHV Mutated CLL
AUC: Area Under the Curve
CR: Complete Response
RBC: Red Blood Cell
CPDA: Citrate phosphate dextrose adenine
ATCC: American Type Culture Collection
DMEN: Dulbecco's Modified Eagle's Medium
BB: Binding Buffer.
