# Supplementary material for: A novel ex vivo high-throughput assay reveals antiproliferative effects of idelalisib and ibrutinib in chronic lymphocytic leukemia
Source: Oncotarget. 2018 May 25;9(40):26019–31. doi: 10.18632/oncotarget.25419 (PMC5995261; doi:10.18632/oncotarget.25419)
Supplement: Supplementary file 1 [file oncotarget-09-26019-s001.pdf]

A novel *ex vivo* high-throughput assay reveals antiproliferative effects of idelalisib and ibrutinib in chronic lymphocytic leukemia

SUPPLEMENTARY MATERIALS

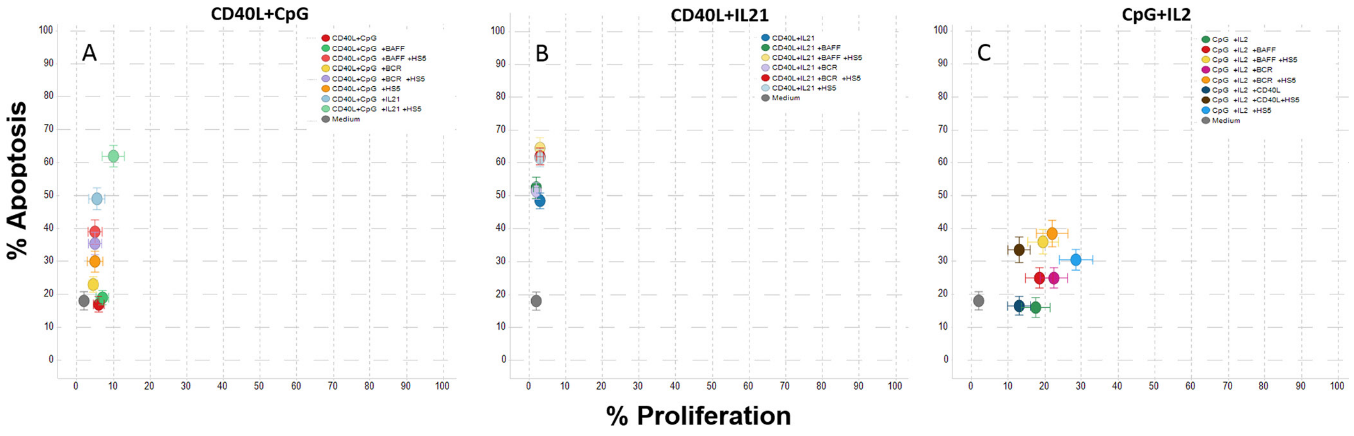

Supplementary Figure 1: Effect of different backbone stimulation on CLL cell survival and proliferation. Results from the 3 backbone stimulation cocktails CD40L+CpG (Panel **A**), CD40L+IL21 (Panel **B**) and CpG+IL-2 (Panel **C**) on 12 progressive samples in terms of survival and proliferation in combination with one or more of the following elements: BAFF, sCD40L, BcR stimulation (anti-IgM) and the HS5 stromal cell line. Results demonstrate CpG+IL2 like the best back bone stimulation.

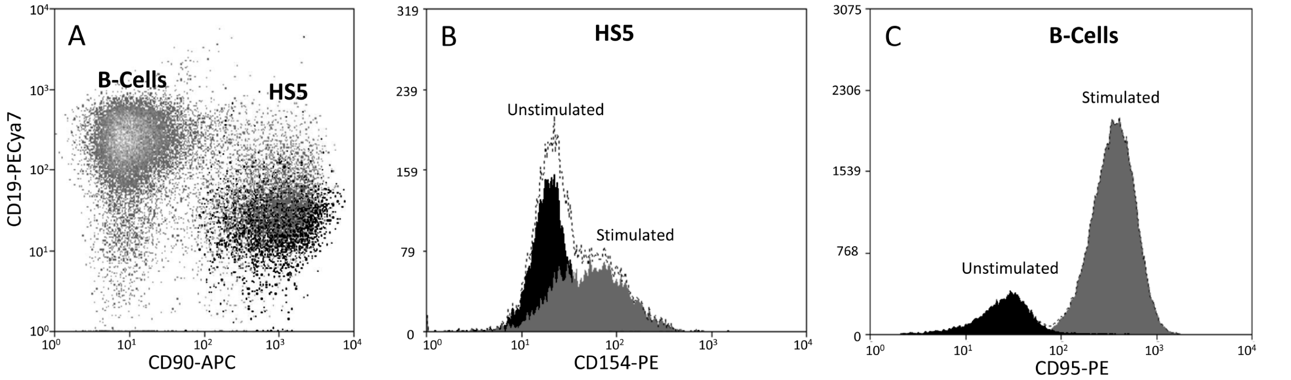

Supplementary Figure 2: Effect of Co-culture on HS-5 cell line and B-Cells. B-Cells (CD19+) and 1:100 HS-5 stromal cell line (CD90+) were incubated alone (black) or in presence (grey) of CpG+IL2+CLL NE (Panel **A**) in the co-culture system. After 96 h hours, HS-5 stromal cell line was trypsinized with trypsin-EDTA and FACS analysis revealed the expression of CD154 (CD40L) in the co-culture and stimulated model (Panel **B**). Non-adherent B-cells were also collected and FACS analysis revealed the expression of CD95 in the co-culture and stimulated model.

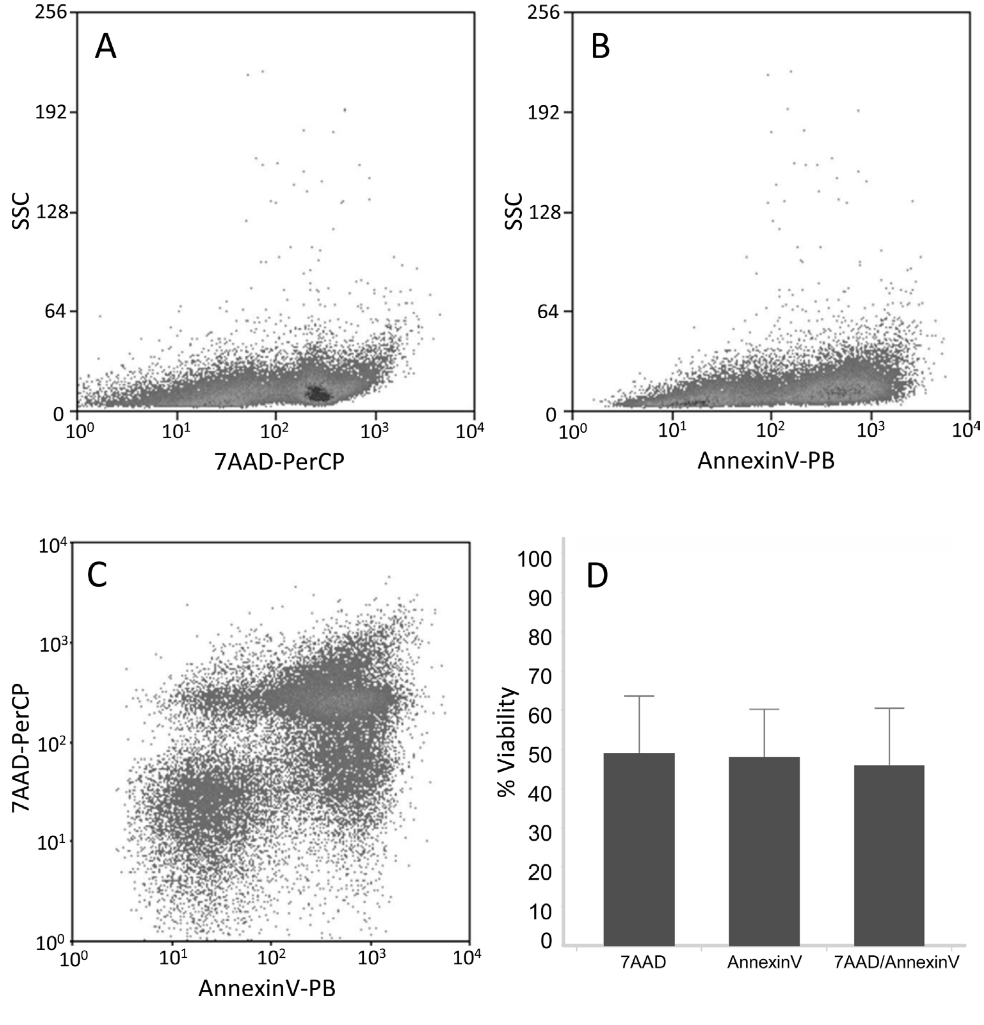

Supplementary Figure 3: Analysis of cell survival by either 7AAD or AnnexinV. Flow cytometry analysis of 96 h incubated B-Cells in presence of different stimulatory factors. Cell survival was evaluated by 7AAD uptake (Panel **A**), AnnexinV (Panel **B**), and both simultaneously (Panel **C**). Live B-Cells exclude 7-AAD and are negative for AnnexinV. Comparison of 7AAD and AnnexinV (Panel **D**) for 12 independent experiments, each performed in triplicate, does not reflect a significant difference between each method.

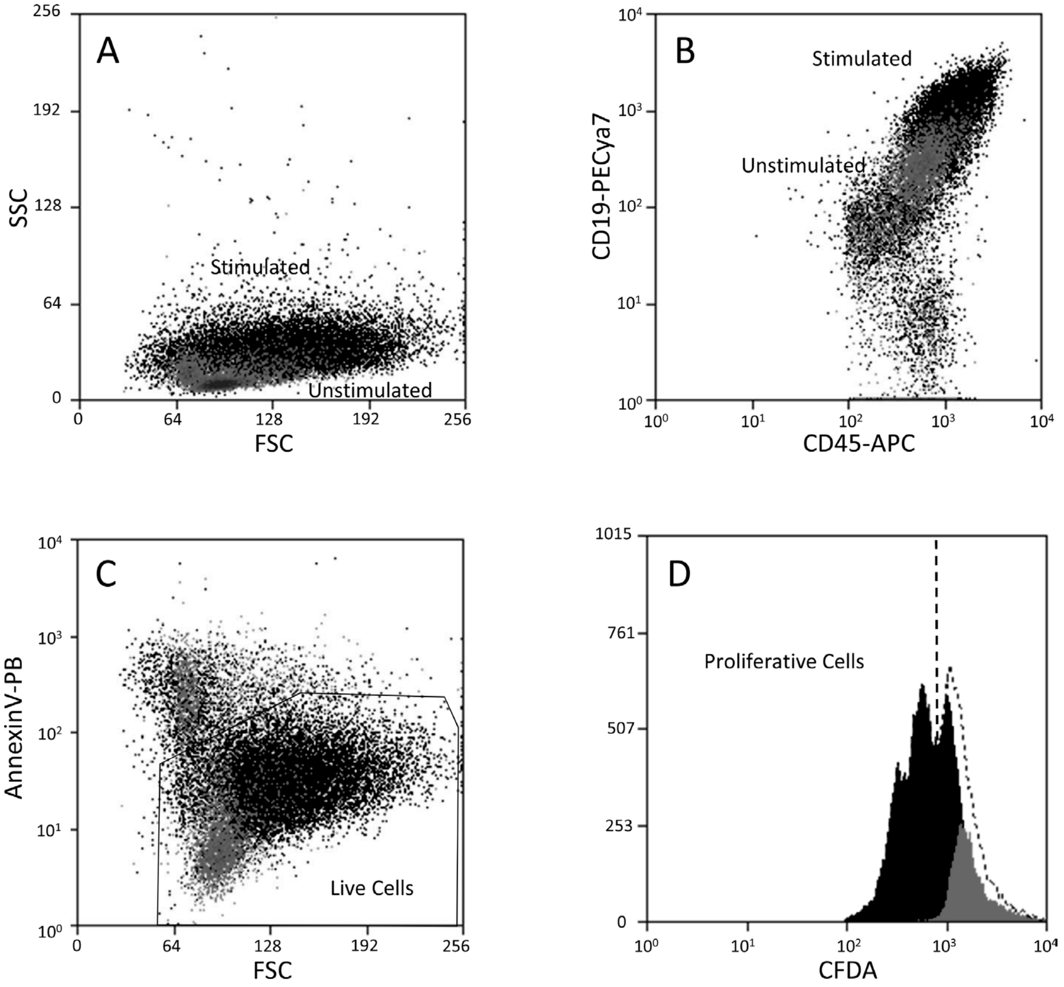

Supplementary Figure 4: Analysis of B-cell survival and proliferation. Illustrative dot plots of the B-cells characteristics and gating strategy for apoptosis and proliferation with (black) or w/o CpG+IL2+CLL+HS5 stimulation (grey). Under this co-culture system, stimulated B-cells increase FSC/SSC (Panel **A**), CD45 and CD19 (Panel **B**). Non apoptotic B-Cells was counted according to the negative AnnexinV staining (Panel **C**) in addition to the absolute cell counting by the PharmaFlow platform. Determination of proliferation in the stimulated condition was determined according to the CFDA fluorescence. Unstimulated cells were used to set the non-division level of CFDA fluorescence and subsequent divisions were considered as proliferative cells (Panel **D**).
